# CD 5+ Peripheral B Cell Lymphoma With Transformation to CD 5+ Diffuse Large B Cell Lymphoma in the CNS: A Case Report Treated With Rituximab, High Dose Methotrexate, Cytarabine, and Intrathecal Methotrexate

**DOI:** 10.7759/cureus.27201

**Published:** 2022-07-24

**Authors:** Dariusz Uczkowski, Emmanuel Apor

**Affiliations:** 1 Internal Medicine, Overlook Medical Center, Summit, USA; 2 Oncology, Overlook Medical Center, Summit, USA

**Keywords:** richter's transformation, high dose methotrexate, r-chop, diffuse large b cell lymphoma, c5+

## Abstract

B-cell non-Hodgkin’s lymphoma includes several subtypes, notably Diffuse Large B-Cell Lymphoma (DLBCL), the most common B-cell subtype. Each presentation has its defining characteristic, of which CD5 positivity is notoriously known for being a poor prognostic factor. CD5 positivity has a high female preponderance, more commonly involves the bone marrow, presents with higher LDH levels and B symptoms on presentation, and stage 3-4 on the diagnosis. The exact incidence of CD5+ DLBCL arising from Chronic Lymphocytic Leukemia (CLL) is not explicitly defined in the literature, but it can be expected in about 5-10% of cases on average. Our patient is a 52-year-old female with no previous history of malignancy who presented with bilateral lower extremity weakness progressing to paraplegia and was found to have a CD5+ B-cell lymphoma in the peripheral blood with a CD5+ Diffuse Large B-cell lymphoma in the central nervous system (CNS). Treatment consisted of Rituximab, High dose Methotrexate (HD-MTX), and Cytarabine/intrathecal Methotrexate for CNS involvement for four cycles. Our patient tolerated therapy with improved neurological symptoms and no evidence of blasts on her peripheral smear or malignant cells on Cerebral Spinal Fluid Flow Cytometry after treatment. Her presentation and response to treatment highlight a possible treatment scheme for this rare and aggressive disease subtype.

## Introduction

The most common subtype of B-cell Non-Hodgkin lymphoma is Diffuse Large B-Cell Lymphoma (DLBCL). Some characteristics of DLBCL include a higher risk for central nervous system (CNS) involvement, systemic symptoms, and the chance of developing CD5 positivity. CD5 positivity is classically associated as a poor prognostic factor, having female preponderance, more bone marrow involvement, higher lactate dehydrogenase levels, B symptoms on presentation, and Ann Arbor stage 3-4 on diagnosis [1]. CD5 positivity in DLBCL has also been associated with Richter’s transformation from CD5+ Chronic Lymphocytic Leukemia (CLL) presenting De-Novo with CD5 markers and rarely acquired during the progression of lymphoma without previous CD5 positivity. Our patient’s presentation was compatible with Richter’s transformation. Due to neurological symptoms being the primary complaint, treatment was favored to involve a CNS approach due to younger age at presentation and having no significant comorbidities. Treatment included Rituximab, High dose Methotrexate, Cytarabine, and intrathecal Methotrexate. Neurologic improvement was appreciated, and no evidence of malignant cells on Cerebral Spinal Fluid Flow Cytometry or peripheral blood flow cytometry after four cycles.

## Case presentation

A 52-year-old-African-American female presents with a four-month history of progressively worsening lower back pain and bilateral lower extremity weakness. Past medical history included hypertension, chronic back pain, and no known prior history of malignancy. On presentation, the patient reported a recent visit to her orthopedic physician with an MRI of the head and lumbosacral spine, with a finding concerning for enhancement of multiple cranial nerves and cauda equina roots suspicious for schwannomatosis. She was subsequently admitted to the hospital and, on presentation, had endorsed a notable 30lb weight loss over the past three months and progressive paraplegia she attributed to her severe back pain.

**Figure 1:**
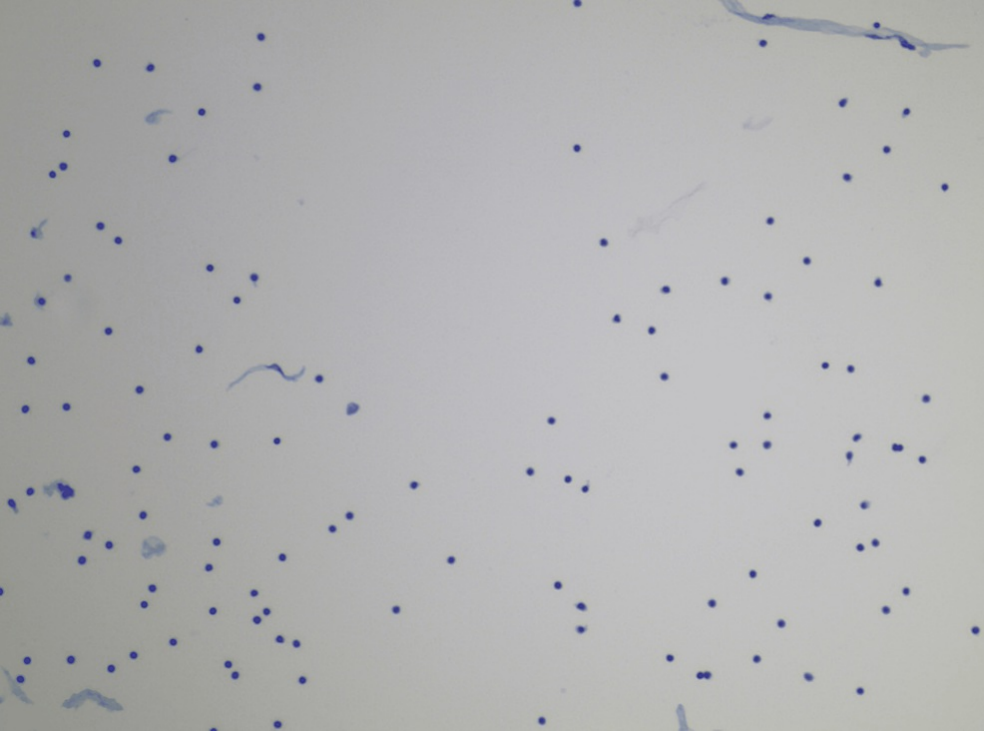
Cerebral Spinal Fluid 100x magnification 100x magnification of Cerebral Spinal Fluid consistent with a small B cell Infiltrate that was CD5+ by flow Cytometry

**Figure 2:**
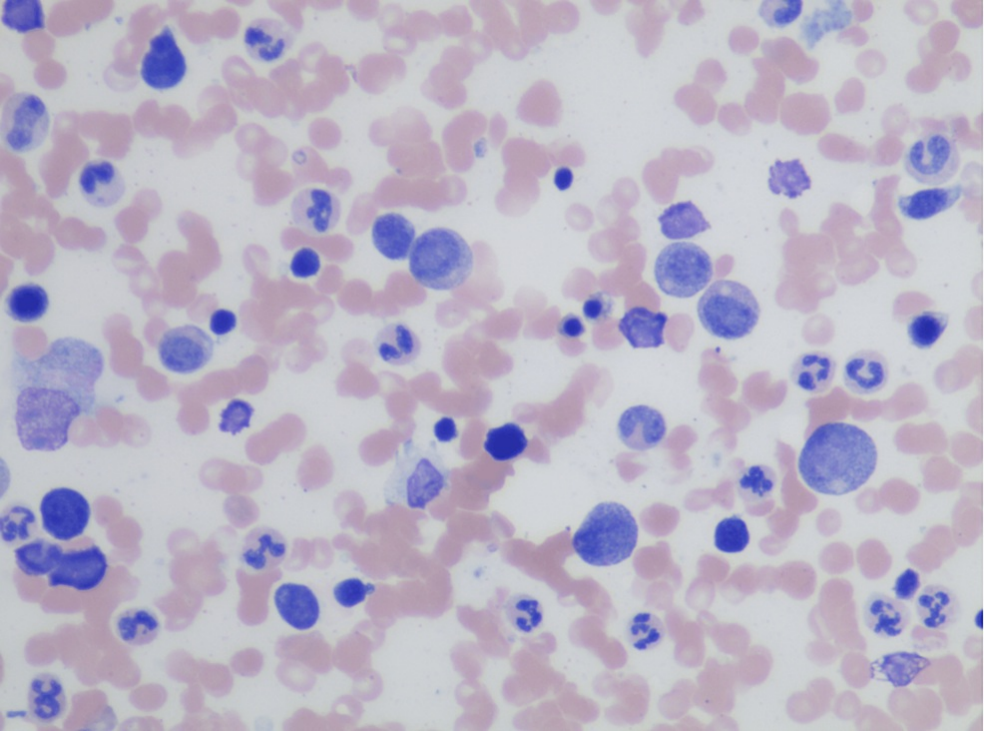
Bone Marrow aspirate 400x magnification 400x magnification of a Bone Marrow aspirate on admission consistent with maturing trilineage hematopoiesis. Few scattered small lymphocytes and no evidence of a large cell infiltrate.

**Figure 3:**
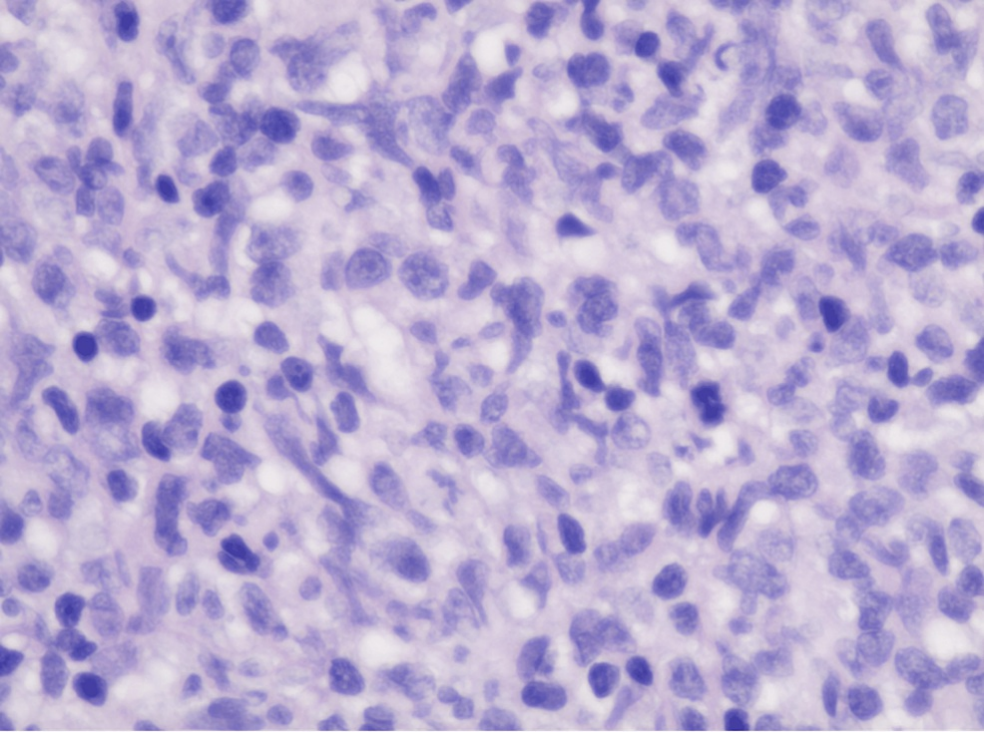
Diffuse Large B-Cell Lymphoma 400x magnification. Diffuse Large B-Cell Lymphoma within the sacral nerve tissue. Polymorphic infiltrate of medium to large lymphoid cells with oval to irregular nuclei, prominent nucleoli and moderate eosinophilic cytoplasm with admixed mitotic figures

**Figure 4:**
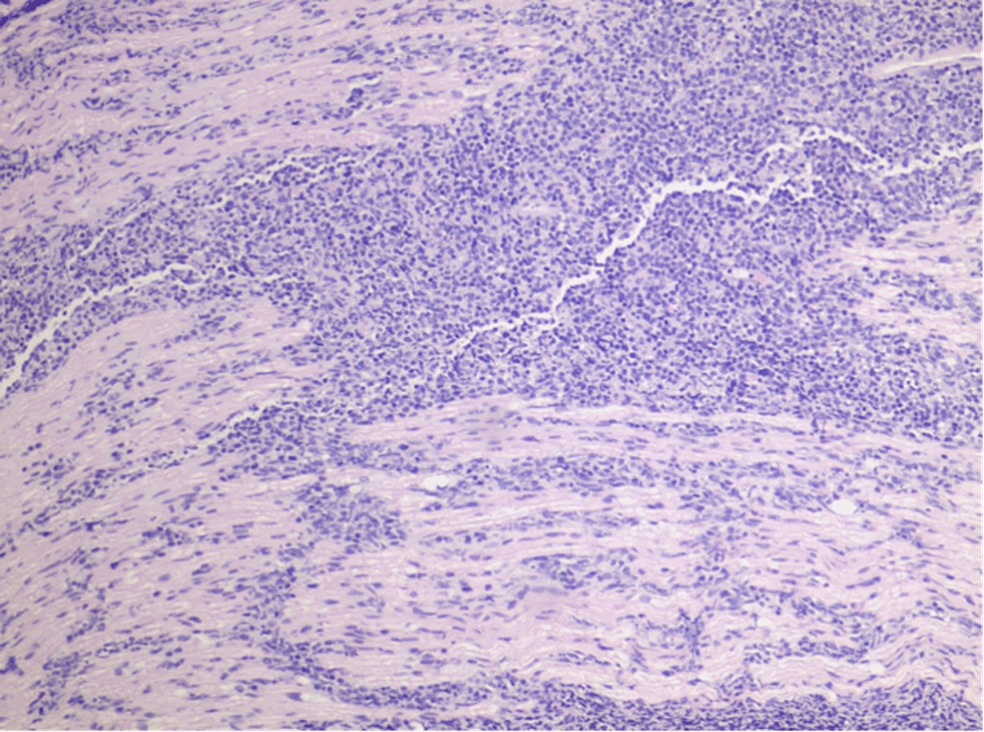
Diffuse Large B-Cell Lymphoma 100x Magnification. A Highlight of the sacral nerve tissue and an infiltrating Diffuse Large-Cell Lymphoma. Moderate eosinophilic cytoplasm is seen again with view primarily highlighting the depth of invasion into the nerve.

MRI of the thorax and cervical spine showed no evidence of cord compression. CT of the abdomen was also unrevealing at the time. Labs included a Hemoglobin of 12 g/dL, White blood cell count of 7.99/nL, Platelets 698/nL, and HIV screen nonreactive. Flow cytometry of peripheral blood was performed and revealed a phenotypic presentation of an Atypical B Cell chronic lymphocytic leukemia (CD5+,CD19+,CD20+,CD22+,CD25+,CD38+, CD45+) (Table 1). A lumbar puncture was performed, which revealed a monoclonal (CD5) B-Cell population (figure 1 and table 2). Pet scan revealed markedly hypermetabolic peripheral nerves involving the cervical region, lumbosacral, and cauda equina. Bone marrow biopsy revealed a minute b-cell population; (-) for large cell lymphoma (Figure 2). Nerve root biopsy was (+) for CD5+ Diffuse Large B Cell Lymphoma (Figures 3, 4). Immunohistochemical staining with Ki-67 revealed a moderately elevated proliferation rate of approximately 50%. The presentation at this time was consistent with a peripheral low-grade-B-cell lymphoma that transformed into a high-grade B-Cell lymphoma in the CNS.

**Table 1:** Peripheral Blood Flow Cytometry Results reported as Negative or Positive if the antibody for the designated Cluster Differentiation (CD) is found CD: Cluster Differentiation, NK cell: Natural Killer Cells, CLL: Chronic Lymphocytic Leukemia, SLL: Small Lymphocytic Leukemia, IL-2: Interleukin 2, sKappa: serum Kappa light chain, sLambda: serum Lambda light chain

Antibody	Description	Results
CD2	Pan T-Cell Antigen	Negative
CD3	Pan T-Cell Antigen	Negative
CD4	T-Helper Subset	Negative
CD5	T-Cell Antigen, Some B-Cell Lymphomas	Positive/Dim
CD7	Pan-T Cell Antigen	Negative
CD8	T-Suppressor Subset	Negative
CD10	Precursor Lymphomas and Leukemias	Negative
CD11c	Monocytes, Hairy Cells	Negative
CD16	Granulocytes, NK cells	Negative
CD19	Early B Cells	Positive/Dim
CD20	Mature B Cells	Positive/Dim
CD22	B-Lymphocytes, Pro-Lymphocytic and Hairy Cell Leukemias	Positive/Dim
CD23	Activated B-Cells, CLL/SLL	Negative
CD25	Activated Cells, IL-2 Receptor, Some T-Cell Lymphomas/Leukemias	Positive/Dim
CD38	Progenitor Lymphoid Cells, Blasts, Plasma Cells	Positive/Dim
CD45	Pan-Leukocyte Antigen	Positive/Bright
CD56	T-Cell Subset, Plasma and NK Cells	Negative
CD57	Neuroendocrine marker, NK cells	Negative
CD103	Hairy Cells, T-Lymphocytes	Negative
sKappa	Plasma Cells, B Lymphocytes	Positive/Moderate
sLambda	Plasma Cells, B Lymphocytes	Negative

**Table 2:** Cerebral Spinal Fluid Flow Cytometry Results are reported as Negative or Positive if the antibody for the designated Cluster Differentiation (CD) is found CD: Cluster Differentiation, NK cells: Natural Killer cells, CLL: Chronic Lymphocytic Leukemia, SLL: Small Lymphocytic Leukemia, sKappa: serum kappa light chain, sLambda: serum lambda light chain

Antibody	Description	Results
CD2	Thymic and peripheral T-cells, NK cells	Negative
CD3	Pan T-Cell Antigen	Negative
CD4	T-Helper Subset	Negative
CD5	T-Cell Antigen, Some B-Cell Lymphomas	Positive/Dim
CD7	Pan-T Cell Antigen	Negative
CD8	T-Suppressor Subset	Negative
CD10	Precursor Lymphomas and Leukemias	Negative
CD16	Granulocytes, NK cells	Negative
CD19	Early B Cells	Positive/Dim
CD20	Mature B Cells	Positive/Dim
CD23	Activated B-Cells, CLL/SLL	Negative
CD38	Progenitor Lymphoid Cells, Blasts, Plasma Cells	Positive/Dim
CD45	Pan-Leukocyte Antigen	Positive/Bright
CD56	T-Cell Subset, Plasma and NK Cells	Negative
CD57	Neuroendocrine marker, NK cells	Negative
sKappa	Plasma Cells, B Lymphocytes	Positive/Dim
sLambda	Plasma Cell, B Lymphocytes	Negative

Due to the patient being mostly symptomatic from her CNS disease, treatment included a CNS lymphoma regimen with Rituximab 500mg/m2, HD-MTX 3,500mg/m2, and Cytarabine 2,000mg/m2 q12 x4 doses. Lumbar puncture was performed after one week of therapy and revealed 42 WBC u/L; flow cytology was (+) for lymphoma. 6mg of Methotrexate was administered intrathecally subsequently after. At the end of Cycle 1, complications included severe cytopenias and neutropenic fever/Klebsiella pneumonia. Cycle 2 included rituximab 500mg/m2, HD-MTX 2,275mg/m2 (dose reduced 35% due to renal dysfunction), Cytarabine 2,000mg/m2 q24 (dose reduced 50% due to sepsis/neutropenic fever) was tolerated better with patient endorsing better mobility and the ability to ambulate with assistance. Lumbar puncture revealed 25 WBC and (-) cytology for lymphoma. Cycle 3 and 4 included: rituximab 500 mg/m2, HD-MTX 3,500mg/m2, Cytarabine 2,000mg/m2 every 24 hours for 2 doses.

**Figure 5:**
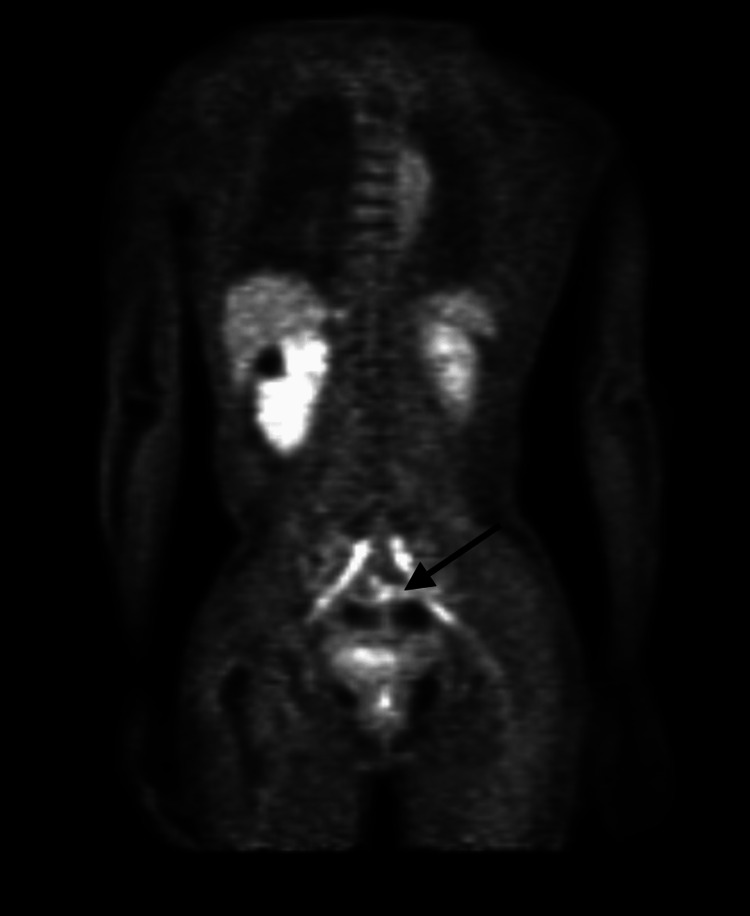
Pre-Chemotherapy PET Scan Arrow highlighting sacral nerve enhancement consistent with the metabolic activity of the Diffuse Large B-Cell Lymphoma

**Figure 6:**
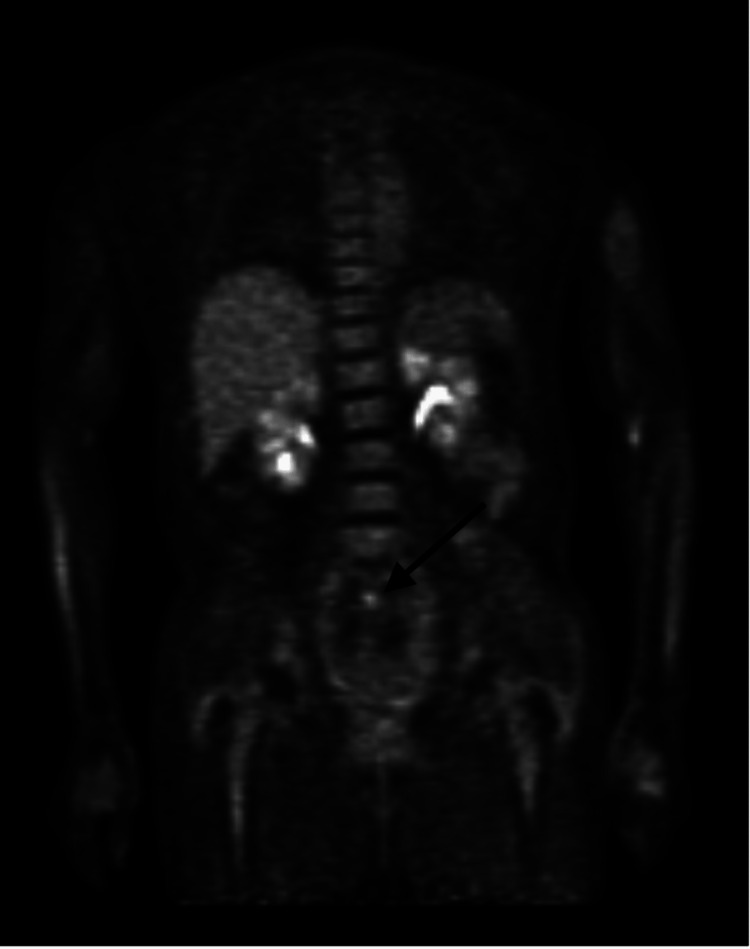
Post Chemotherapy PET Scan Arrow highlighting interval improvement of sacral nerve involvement of Diffuse Large B-Cell Lymphoma after Chemotherapy on PET Scan

Lumbar puncture after cycle four was (-) for malignant cells via CNS flow cytometry, and peripheral smear was also (-) for lymphoma cells. The PET scan showed no hypermetabolism areas besides the physiologic areas (Figures 5, 6). The patient significantly improved her mobility and was able to ambulate with assistance. The patient was then referred for an autologous stem cell transplant and remained in remission of her disease.

## Discussion

DLBCL with CD5 positivity is notoriously known as an aggressive subset, with most studies arising primarily from Japan and recent case reports highlighting this subset. The characteristics of CD5(+) patients include high female preponderance, high incidence of CNS involvement, more commonly involving the bone marrow, higher LDH levels, B symptoms on presentation, and advanced Stage 3-4 on diagnosis [1]. The exact incidence of C5+ Diffuse large B-cell lymphoma arising from CLL is not explicitly defined in the literature. Still, it can be roughly estimated to be about 5-10% of cases on average based on data available in the literature review [1]. The mechanism proposed for CD5 positivity expression is likely related to the facilitated migration of lymphocytes from lymph nodes into the systemic circulation [2,3]. CD5 CLL cells, in particular, have also been shown to promote the malignant CLL phenotype, thus promoting metabolic pathways and proliferation [4]. This cluster differentiation also stimulates the release of IL-10, which is proposed to exert negative feedback mechanisms leading to the prevention of B cell death [3].

Historically, treatment schemes for CD5+ DLBCL were limited to a few studies that identified regimens such as CHOP (cyclophosphamide, doxorubicin hydrochloride, vincristine, prednisone) with or without Rituximab. Yamaguchi et al. and Miyazaki et al. from Japan compared overall survival when comparing chemotherapy with versus without Rituximab [5,6]. Notably, Yamaguchi et al. identified cases of De-Novo CD5+ DLBCL and administered an anthracycline without a rituximab regimen. 63% Complete remission (CR) was observed in 69/109 patients and 5-year overall survival (OS) of 34% [5]. Miyazaki et al.’s retrospective study included 337 CD5+ DLBCL patients and excluded Intravascular B-cell lymphoma, Primary DLBCL of the CNS, and secondary CD5+ DLBCL [6]. In total, 184 patients received R-CHOP, and 153 received CHOP alone. 80% CR was seen in the R-CHOP group and 66% CR in the chemotherapy alone group [6]. Overall survival at two years was 70% with R-CHOP and 54% with CHOP [6].

More recently, Nelson et al. noted a reduction in blast count and leukocytosis with an R-CHOP, intrathecal Methotrexate, and cytarabine regimen. Their patient notably had CNS involvement on presentation, and pathology was most consistent with a stage 3B follicular lymphoma that transformed to a CD5+ DLBCL [7]. Although differences between De-novo CD5+ DLBCL, Richter’s syndrome associated DLBCL, and secondary CD5+ DLBCL are all pathogenetically distinct, each previously highlighted treatment regimen still requires further clinical trials to determine the optimal treatment scheme for CD5+ DLBCL as a subset [8,9]. Additionally, our regimen with Rituximab, High Dose Methotrexate, Cytarabine, and Intrathecal Methotrexate improved neurological symptoms, absence of peripheral blasts, and negative CNS cytology also requires dedicated clinical trials.

## Conclusions

In conclusion, our report illustrated an unusual presentation of lymphoma with paraplegia as the presenting symptom. Awareness for aggressive subtypes such as CD5+ DLBCL can lead to a timely diagnosis for this subset which unfortunately has an overall poor prognosis. Cases of CD5+ high-grade lymphomas have been reported, but historically, outcomes have not been favorable. Our case was unique as our patient presented with an atypical CD5+ CLL with transformation to CD5+ DLBCL with a favorable response to Rituximab, High Dose Methotrexate, Cytarabine, and Intrathecal Methotrexate, which to our knowledge is one of the few reported cases to effectively treat CNS relapse and time to autologous stem cell transplant for curative intent in this subset.
